# Use of detailed family history data to improve risk prediction,with application to breast cancer screening

**DOI:** 10.1371/journal.pone.0226407

**Published:** 2019-12-17

**Authors:** Yue Jiang, Clarice R. Weinberg, Dale P. Sandler, Shanshan Zhao

**Affiliations:** 1 Biostatistics and Computational Biology Branch, National Institute of Environmental Health Sciences, Research Triangle Park, North Carolina, United States of America; 2 Department of Biostatistics, University of North Carolina, Chapel Hill, North Carolina, United States of America; 3 Epidemiology Branch, National Institute of Environmental Health Sciences, Research Triangle Park, North Carolina, United States of America; University of South Alabama Mitchell Cancer Institute, UNITED STATES

## Abstract

**Background:**

As breast cancer represents a major morbidity and mortality burden in the U.S., with about one in eight women developing invasive breast cancer over her lifetime, accurate low-cost screening is an important public health issue. First-degree family history, often simplified as a dichotomous or three-level categorical variable (0/1/>1) based on number of affected relatives, is an important risk factor for many conditions. However, detailed family structure information such as the total number of first-degree relatives, and for each, their current or death age, and age at diagnosis are also important for risk prediction.

**Methods:**

We develop a family history score under a Bayesian framework, based on first-degree family structure. We tested performance of the proposed score using data from a large prospective cohort study of women with a first-degree breast cancer family history. We used likelihood ratio tests to evaluate whether the proposed score added additional information to a Cox model with known breast cancer risk factors and the three-level family history variable. We also compared prediction performance through Receiver Operating Characteristic (ROC) curves and goodness-of-fit testing.

**Results:**

Our proposed Bayesian family history score improved fit compared to the commonly used three-level family history score, both without and with adjustment for other risk factors (likelihood ratio tests p = 0.003 without adjustment for other risk factors, and p = 0.007 and 0.009 under adjustment with two candidate sets of risk factors). AUCs of ROC curves for the two models were similar, though in all cases were higher after addition of the BFHS.

**Conclusions:**

Capturing detailed family history data through the proposed family history score can improve risk assessment and prediction. Such approaches could enable better-targeted personalized screening schedules and prevention strategies.

## Introduction

The American Cancer Society recommends routine screening mammography for women above age 45 [1]. However, concerns persist regarding the potential for over-diagnosis among low risk women [2]. At the same time, it is beneficial to identify women at unusually high risk who would most likely benefit from screening and early preventive interventions such as risk-lowering drugs, with accurate risk prediction tools enabling better-personalized prevention. Several important demographic, lifestyle, and genetic risk factors for breast cancer have been identified [3], including age, age at menarche, first live birth, and menopause, prior breast biopsy, obesity, alcohol consumption, hormone therapy, and germline BRCA1 and BRCA2 mutations. Various risk prediction tools have been developed based on these risk factors; among them, the Gail breast cancer risk prediction model [4] is widely used in clinical practice for population screening as the risks are calculated based on demographic, family history, and lifestyle factors only, which are all easy to obtain. BOADICEA [5], BRCAPRO [6] and the iCARE package [7] improve the Gail model by additionally including pedigree and genetic information. However, some of this information is not easily captured, and thus these models are more suitable for genetic counseling or for facilitating medical diagnosis.

Family history is well-known as an important risk factor for breast cancer, reflecting joint effects of environment, lifestyle, and genetics. A woman’s breast cancer risk doubles if she has at least one first-degree female relative with breast cancer, with higher relative risk if she is younger and/or if affected relatives were diagnosed at a young age [8,9]. Studies also suggest increased risk as the number of affected first-degree female relatives increases, with similar risk implications for affected mothers vs. sisters [8–10]. The number of affected family members and their ages are known to make a difference. For instance, a woman with four out of six sisters with breast cancer is at higher risk than a woman with only two out of six. A woman with four elderly cancer-free sisters is at lower risk than a woman with four young cancer-free sisters, since the young cancer-free sisters are still at risk for developing breast cancer over their lifetimes whereas the elderly sisters definitely did not develop it.

**Fig 1:**
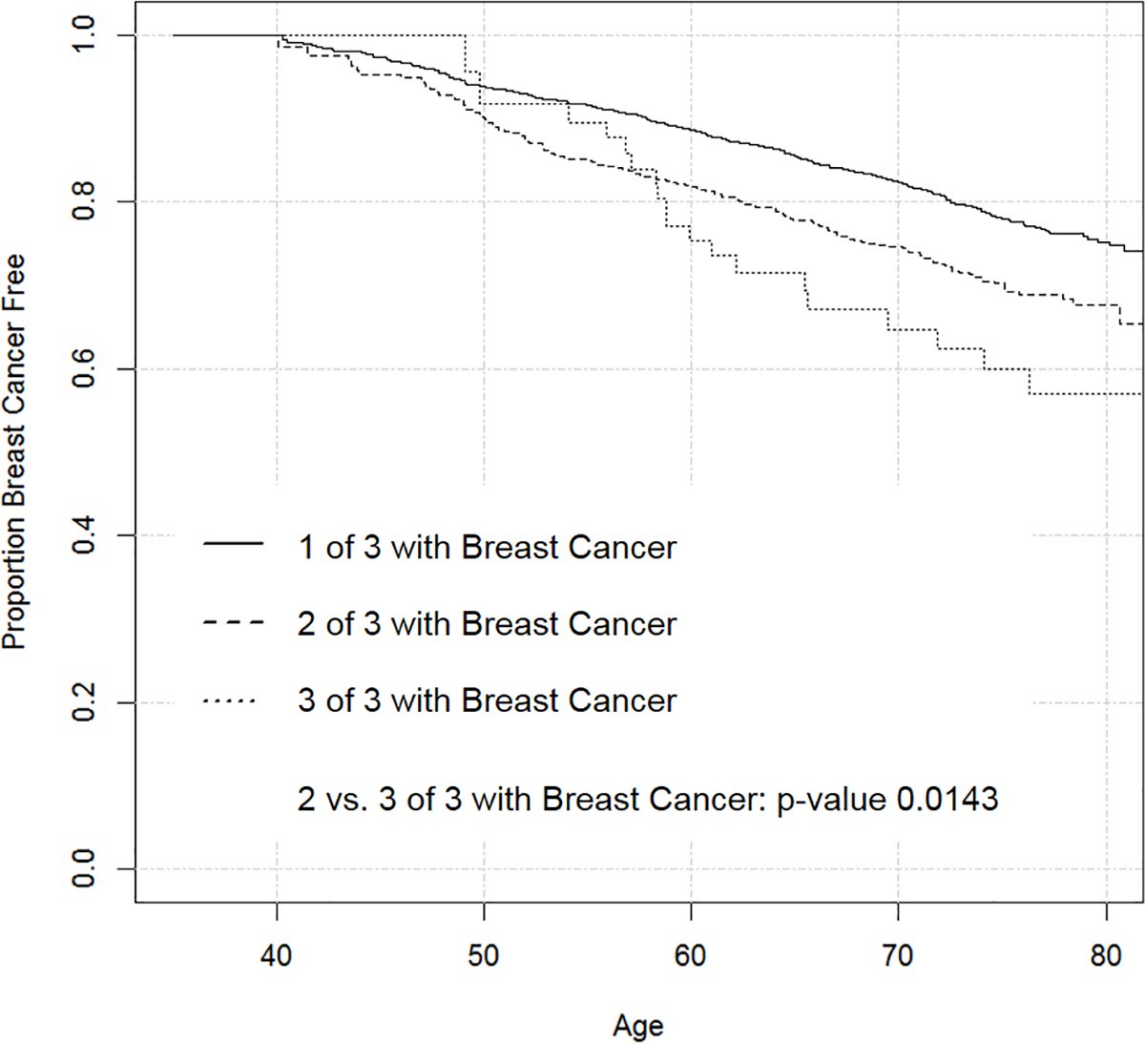
Kaplan-Meier curves of proportion breast-cancer-free for Sister Study participants with three first-degree female relatives, stratified by number of first-degree female relatives with breast cancer.

Although these aspects of family history are known to correlate with breast cancer risk, in many prediction models, detailed family history information is simplified into a single categorical variable based on the number of breast cancer cases among first-degree female relatives. The Gail model uses a three-level categorical variable (0/1/>1) that could lead to loss of predictive power compared to more informative (but harder to ascertain) scores. In S1 Fig, we display Lexis diagrams of two hypothetical families with the same structure and would both be in the >1 category with the three-level family history score, but the first family would be at higher risk with a larger number of BCa cases and younger age at onset. This also can be illustrated in the prospective NIEHS Sister Study [11]: In Fig 1, we plot Kaplan-Meier curves for the probability of remaining breast cancer-free in a subcohort of the Sister Study that was selected among participants with exactly three first-degree female relatives. There are statistically significant differences in breast cancer risk between those with 2 vs. 3 affected relatives (log-rank test, *p* = 0.0143), a distinction obscured by using a single “>1” category.

Our objective was to develop a risk assessment tool that quantifies such differences by better incorporating details of family structure and history while still being based on easily-collected information that can be used for wider population screening. We focus on first-degree relatives because reporting for them is likely to be more accurate and complete than that for second-degree relatives, even though a full pedigree might contribute additional information.

## Methods

### The sister study

We used data from The Sister Study (https://sisterstudy.niehs.nih.gov), a prospective study of women with positive family history of breast cancer [11]. During 2003 to 2009, 50,884 breast cancer-free women aged 35–74 from the U.S. and Puerto Rico with at least one affected full or half sister were enrolled. At baseline, family breast cancer history, demographics, and information on relevant lifestyle and reproductive risk factors were collected. Breast cancer diagnoses, including invasive and ductal carcinoma *in situ* (DCIS), were self-reported and confirmed using medical records. We focused on non-Hispanic white women only in this analysis, restricting family history information to first-degree female relatives in order to best demonstrate comparisons to existing, well-validated methods that use the simple 0/1/>1 score. For families with multiple participating women, we randomly selected one to avoid dependencies. About 6.5% of Sister Study participants reported having half-sisters. For the purpose of our analysis, half-sisters were considered second-degree relatives thus omitted from consideration. Our final analysis cohort consisted of 37,720 women, of whom 814 (2.2%) reported only a half-sister with breast cancer and thus had no affected first-degree female relatives.

### Bayesian family history score

As preliminary definitions, it is important to distinguish between pure and absolute risk. Absolute risk is the probability that a disease-free individual will develop the disease of interest within some interval. This is the risk of experiencing the event of interest in the presence of competing risks (for instance, dying of non-breast cancer related causes), and mimics the real-world setting. On the other hand, pure risk is defined to be the probability of developing the disease of interest in the absence of all other competing risks (for instance, as estimated by a Cox model). For a detailed explanation and illustrative example, consult Gail [12].

We assume that for each family, the lifetime *pure* family-specific breast cancer risk *p* follows a *Beta*(*α*,*β*) prior distribution, with parameters satisfying two constraints. First, the expected value of the population *pure* lifetime breast cancer risk (comprising both invasive and *in situ* cancers), *p*_0_ = *α*/(*α*+*β*), was set to 0.197 based on a calculation from Surveillance, Epidemiology, and End Results (SEER) (April 2017 Data Release) cancer registry data [13] whereby we integrated the estimated one-year breast cancer hazards and worked backward to estimate the survivor function. As expected, this is higher than the *absolute* lifetime risk of invasive and *in situ* breast cancer of 0.149 based on the same SEER data. Second, the pure lifetime risk of breast cancer given an affected first-degree female relative was assumed to be double compared to a cancer-free family [8,9]. These two constraints lead to exact values for *α* and *β* as 1.184 and 4.828, respectively. We further assume the family-specific hazard function is *λ*_*p*_(*t*) = *f*(*p*)*λ*_0_(*t*), where *t* denotes age, *λ*_0_(*t*) is the population baseline hazard, and the effect of family history is multiplicative through *f*(*p*). With this information, we can fully specify the likelihood.

For a specific family with *n* first-degree female family members, we observe (*X*_*i*_,*d*_*i*_) for the i^th^ female relative, i = 1, 2,…,n, where *X*_*i*_ = min(*T*_*i*_,*C*_*i*_) is the minimum of her censoring time (*C*_*i*_) (age time at which we ascertain her status) and age time of breast cancer onset (*T*_*i*_), and *d*_*i*_ = *I*(*X*_*i*_ = *T*_*i*_) is an indicator of whether she was diagnosed with breast cancer. Through the likelihood, we incorporate breast cancer history of family members up to their current age, death age, or diagnosis age, with their breast cancer experience being weighted by their aggregated risks up to their censoring or diagnosis ages.

We use the posterior expectation of the family-specific lifetime risk $\hat{p}$ as our Bayesian Family History Score (BFHS):

$$\hat{p}=E(p;X,d)=\frac{\int_{0}^{1}p^{\alpha }{\left(1-p\right)}^{\beta -c-1}{\left(\log\left(1-p\right)\right)}^{\sum_{i=1}^{n}d_{i}}dp}{\int_{0}^{1}p^{\alpha -1}{\left(1-p\right)}^{\beta -c-1}{\left(\log\left(1-p\right)\right)}^{\sum_{i=1}^{n}d_{i}}dp}.$$

Here, $c=\sum_{i=1}^{n}\frac{\int_{0}^{X_{i}}\lambda_{0}(t)dt}{\log\left(1-p_{0}\right)}$ is a unique family-specific parameter that accounts for both family size and total risk experienced. Full details regarding derivation of BFHS are provided in S1 Appendix.

One advantage of our score is that $\hat{p}$ is directly interpretable as the pure family-specific lifetime breast cancer risk. This continuous estimate can be plugged in directly to calculate age-specific risks through *λ*_*p*_(*t*). Our score can be used as a covariate (e.g., in a logistic regression or Cox model), together with other variables, to represent the family structure and history. Finally, this score is easily updated as time passes and further breast cancer cases occur, enabling improved future prediction.

### Comparison of methods for accounting for family history

To compare the three-level family history score (0/1/>1) and our proposed Bayesian family history scores (BFHS), we focus on three sets of paired Cox models with time-to-breast cancer as the outcomes. In each pair, model (a) uses the three-level 0/1/>1 score as the family history covariate (in conjunction with specified other known breast-cancer covariates), and model (b) *additionally* includes the BFHS. A likelihood ratio test is performed on these nested Cox models in order to determine whether inclusion of the BFHS significantly improves the fit of the model in the presence of the particular covariates. The covariates used in these three sets of models are as follows:

1. Model 1: family history variable(s) only
2. Model 2: family history variable(s), Gail model-identified covariates and interactions
3. Model 3: family history variable(s), Gail model-identified covariates and interactions, and baseline menopause status and BMI category

Table 1 details which covariates and interactions are found in each model.

**Table 1:** Models to be compared.

	*Model 0*	*Model 1*		*Model 2*		*Model 3*	
		1a	1b	2a	2b	3a	3b
*0/1/>1 Family History Score*	**x**	**x**	**x**	**x**	**x**	**x**	**x**
*Bayesian Family History Score (BFHS)*			**x**		**x**		**x**
*Age at Menarche (Categorical)*	**x**			**x**	**x**	**x**	**x**
*Total Biopsies (Categorical)*	**x**			**x**	**x**	**x**	**x**
*Age at First Life Birth^**a**^ (Categorical)*	**x**			**x**	**x**	**x**	**x**
*Age >50 Indicator*	**x**			**x**	**x**	**x**	**x**
*BMI (Categorical)*						**x**	**x**
*Menopause Status at Baseline*						**x**	**x**
*Interaction:* *Age >50 Indicator* *by Total Biopsies (Categorical)*	**x**			**x**	**x**	**x**	**x**
*Interaction:* *0/1/>1 Family History Score* *by Age at First Live Birth^**a**^ (Categorical)*	**x**			**x**	**x**	**x**	**x**

^**a**^ Age at first live birth categories grouped ages 25–29 and nulliparous women for Models 0, 1a, 1b, 2a, and 2b. For Models 3a and 3b, these were treated as separate categories.

The detailed covariate definition in Models 2 and 3 are slightly different from the original Gail model as follows. Model 2 adjusts for known breast cancer risk factors as in the Gail model: baseline age (<50 vs. ≥50), age at menarche (<12, 12–13, ≥14), number of prior biopsies (0, 1, ≥2), age at first live birth (<20, 20–24, 25–29 (or nulliparous), or ≥30), an interaction between baseline age and number of biopsies, and an interaction between age at first live birth and three-level family history. In the original Gail model, all categorical risk factors were treated linearly, with risk ratios assessed proportionally according to ordering. However, given the large sample size of the Sister Study, we were able to include these covariates and interactions categorically. A likelihood ratio test suggested that using them categorically provided a better fit for our data (p = 0.003). Accordingly, all models considered in this analysis used categorical covariates and interactions to best utilize available data. Model 3 included the additional covariates of baseline menopause status and BMI category, which have been shown in prior studies to be significantly associated with breast cancer risk [14,15], and were additionally selected in our cohort by forward model selection. Additionally, in Model 3, we separated nulliparous women from women aged 25–29 at their first live birth into two categories, as we found that this resulted in better fit in our cohort.

As our cause-specific Cox models estimate pure risks and are thus subject to the competing risk of death, to make a fair comparison to the Gail model, we must estimate absolute risks based on our data. We first estimated age-specific mortality rates by using a LOESS smoothed curve on Sister Study data. As the Sister Study population is healthier than the population at large, the estimated mortality rates are generally lower than equivalent national mortality rates; 363 (1.0%) participants died within five years without having developed breast cancer. We then calculate the absolute risk with an approach similar to the one by Gail et al. in prior analysis [4] using the estimated mortality rate and the estimated pure risk from the Cox models. Detailed methodology regarding the calculation of these absolute risks is provided in S2 Appendix. In the absence of similar data, one may use national age-specific mortality data, for instance as published by the National Vital Statistics System [16].

We further compared the prediction performance of the Bayesian score with the three-level family history score by using these calculated five-year absolute risks for Models 1, 2, and 3, comparing models in which the family history variable used was the three-level 0/1/>1 variable to those in which we additionally included the BFHS. We binned women into 1% risk categories ranging from 1% (or lower) to 10% (or higher) according to the calculated five-year absolute risks, and plotted bar graphs of the observed number of women in each category who developed breast cancer within five years against the expected number as calculated by the candidate models. We additionally provided 95% exact binomial confidence intervals for each bin. To assess overall goodness-of-fit, a χ^2^ statistic was calculated as

$$\chi_{9}^{2}=\sum_{k=1}^{10}\frac{{\left(O_{k}-(\frac{k}{100})\times n_{k}\right)}^{2}}{(\frac{k}{100})\times n_{k}},$$

where *k* = 1, 2, …, 10 indexes the risk bins, *O*_*k*_ denotes the observed number of breast cancer cases, and *n*_*k*_ the total number of women assigned to bin k by the model (*kn*_*k*_/100 being the expected number of breast cancer cases, noting the slight approximation incurred by using 10% for the 10% or higher bin). There were 853 (2.3%) women who were lost to follow-up prior to five years without dying or developing breast cancer in the five year interval. For these women, we weight their contribution to the denominator by the ratio of the absolute risk they experienced during follow up to their five-year absolute risk. For our purposes, a *less significant* p-value indicates better fit. As a base comparator, we examined the performance of the Gail model itself (Model 0) as implemented by the Breast Cancer Risk Assessment Tool, available online from NCI (https://www.cancer.gov/bcrisktool/).

For the above goodness-of-fit calculations we directly estimated the baseline hazard from our data given that our cohort of women with positive family history is likely at a higher risk of breast cancer; however, in the absence of this contextual information, one can estimate the baseline hazard using SEER data. To avoid overfitting on our dataset during this comparison, we utilized ten-fold cross-validation to fit these models using the same training and testing datasets for all sets of models.

To evaluate the predictive performance of each model, receiver operating characteristic (ROC) analysis was also performed on the calculated five-year absolute risks. As these tests can potentially be used as screening tools for the general population, we focused on thresholds that correspond to 90% or greater specificity, to limit false positives. We calculated area under the curve (AUC), partial area under the curve (pAUC) and sensitivities. We did not have sufficient numbers of low risk women to evaluate low-risk thresholds due to the nature of our study participants.

## Results

Among the 37,720 participants used for analysis, 2,181 (5.8%) developed breast cancer through July 2017 (Data Release 6.0), including both invasive breast cancer and DCIS. 1,352 of these cases were diagnosed within 5 years of enrollment. On average, women had 3.9 (range: 0–16) first-degree female relatives, including 1.3 (range: 0–5) breast cancer cases at baseline; the median follow-up time was 7.89 years (range: 0.01–11.62). Full baseline characteristics are summarized in Table 2.

**Table 2:** Baseline characteristics for non-Hispanic white Sister Study participants.

	*Breast Cancer Free (n = 35,539)*			*Breast Cancer Affected (n = 2,181)*			*p-value^a^*
	N	%	Mean (SD)	N	%	Mean (SD)	
*Number of 1^st^ degree female relatives*			3.92 (1.7)			3.90 (1.7)	0.641
*Number of 1^st^ degree female relatives with breast cancer*			1.27 (0.56)			1.39 (0.62)	<0.001
*Age at baseline*			56.2 (9.0)			57.5 (8.9)	<0.001
*Age at baseline*							<0.001
*<50*	9393	26.4		478	21.9		
*≥50*	26146	73.6		1703	78.1		
*Age at menarche*							0.009
*7–11*	6786	19.1		444	20.4		
*12–13*	20210	56.9		1274	58.4		
*≥14*	8543	24.0		463	21.2		
*Age at first live birth*							0.201
*<20*	4098	11.5		242	11.1		
*20–24*	11325	31.9		684	31.4		
*25–29*	8338	23.5		482	22.1		
*≥30*	5263	14.8		356	16.3		
*Nulliparous*	6515	18.3		417	19.1		
*Previous biopsies*							<0.001
*0*	24204	68.1		1282	58.8		
*1*	4847	13.6		335	15.4		
*>1*	6488	18.3		564	25.9		
*First-degree female relatives with BCa*							<0.001
*0*	783	2.2		31	1.4		
*1*	25497	71.7		1386	63.5		
*>1*	9259	26.1		764	35.0		
*BMI Category*							0.017
*<25*	14770	41.6		840	38.5		
*25–30*	11071	31.2		704	32.2		
*>30*	9698	27.3		637	29.2		
*Baseline Menopause*							0.004
*Pre-Menopausal*	11549	32.5		643	29.5		
*Post-Menopausal*	23990	67.5		1538	70.5		
*Bayesian Family History Score (BFHS)*			0.317 (0.06)			0.328 (0.07)	<0.001

^a^ *p* values were obtained from t-tests for continuous variables and chi-square tests for categorical variables, as appropriate.

814 (2.2%) women had only half-sisters with breast cancer (i.e., had no first-degree female relatives with breast cancer), 26,883 (71.3%) had exactly one first degree female relative (full sister or mother) with breast cancer, and 10,023 (26.6%) had at least two first degree female relatives with breast cancer. Among these three groups, the mean (SD) BFHS were 0.177 (0.01), 0.288 (0.02), and 0.401 (0.04), respectively (ANOVA, p <0.001). When restricting to participants with exactly three first-degree female relatives, as in Fig 1, the BFHS was 0.293 (0.01), 0.408 (0.02), and 0.497 (0.02), for women with 1, 2, and 3 of 3 relatives with breast cancer, respectively (ANOVA; p-value <0.001). Linear trend tests of these ANOVAs were significant (p < 0.001 for both). Sister Study participants who did not develop breast cancer during follow-up had a mean BFHS of 0.317 (0.06), compared to 0.328 (0.07) for women who developed breast cancer during follow-up (p <0.001).

Adding the BFHS to a univariable model that used only the 0/1/>1 family history score significantly improved the fit of the Cox model (Model 1a vs. Model 1b, likelihood ratio test p = 0.003). This conclusion also held in multivariable models that adjusted for Gail-identified risk factors (Model 2a vs. Model 2b, p = 0.007), and in the model that included additional covariates and interactions (Model 3a vs. Model 3b, p = 0.009). By contrast, in the “reverse” analysis in which we instead considered adding the three-level categorical family history to Cox models that already included the BFHS, the fit was not significantly improved (p = 0.4962, p = 0.6665, and p = 0.6278 for Models 1, 2, and 3, respectively).

**Fig 2:**
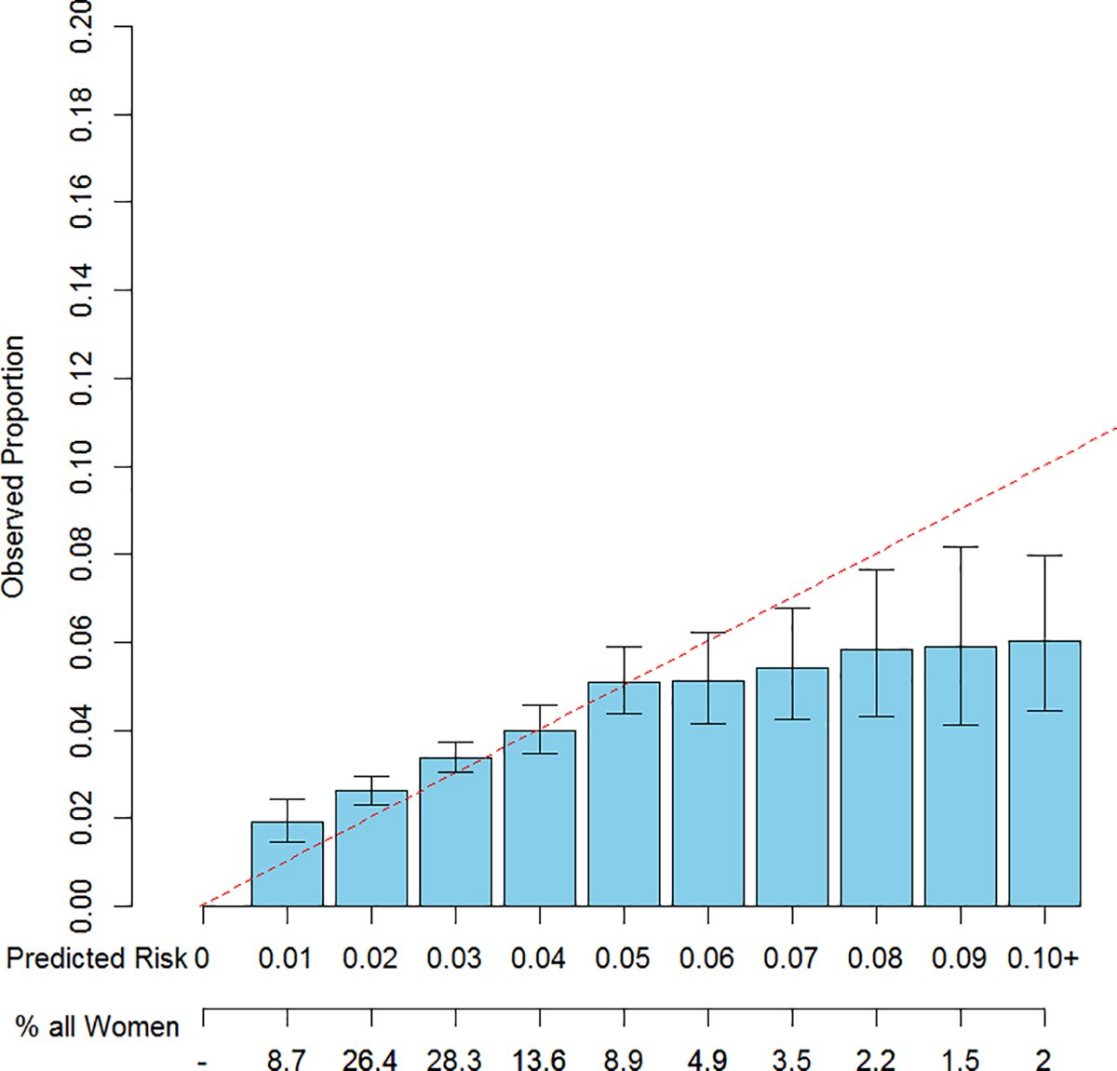
Observed vs. expected counts of 5-year absolute risks vs. 1% risk bins, of Gail Model predicted risks.

**Fig 3:**
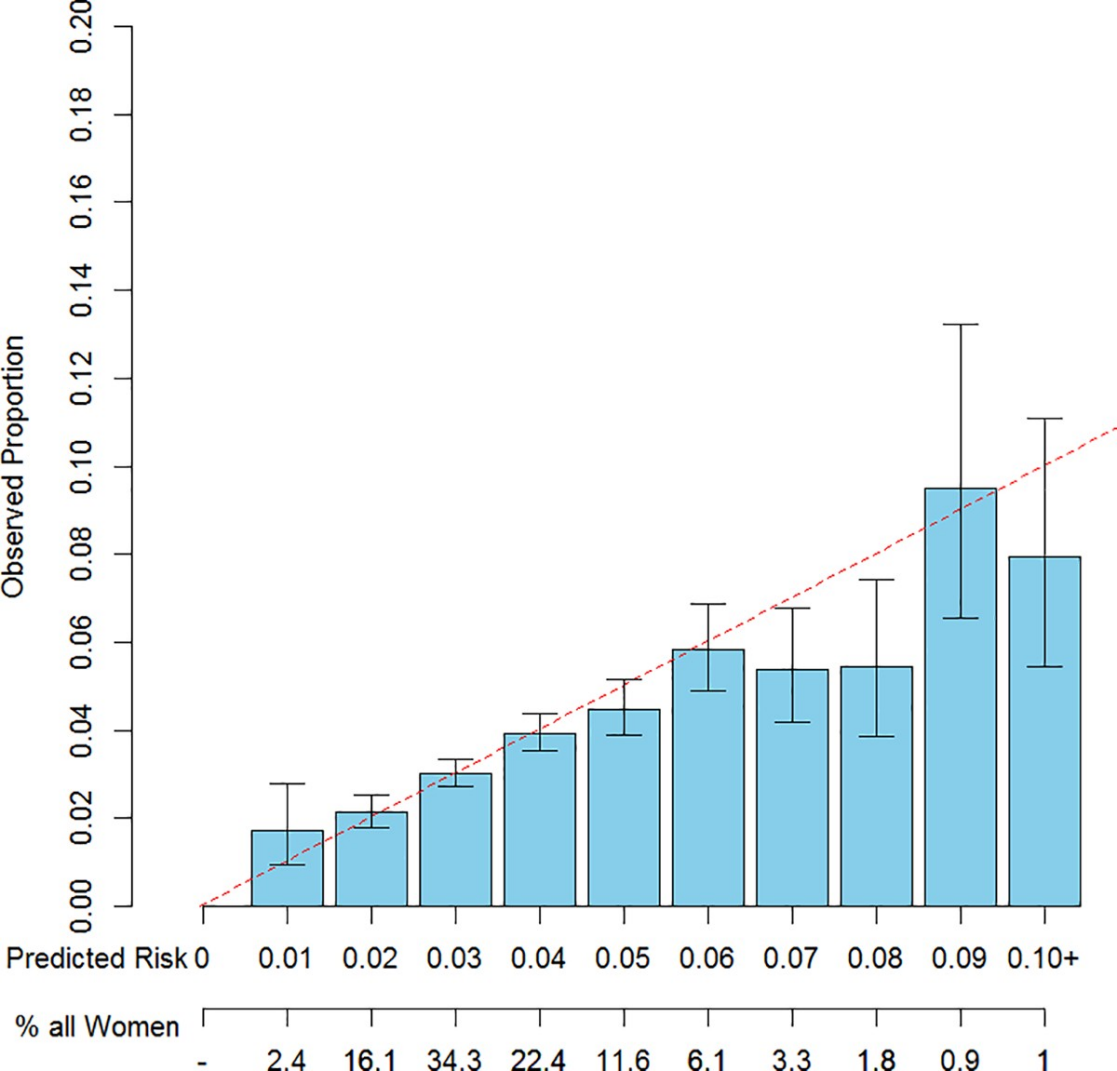
Observed vs. expected counts of 5-year absolute risks vs. 1% risk bins, of 10-fold cross-validated predicted risks using Model 3a.

**Fig 4:**
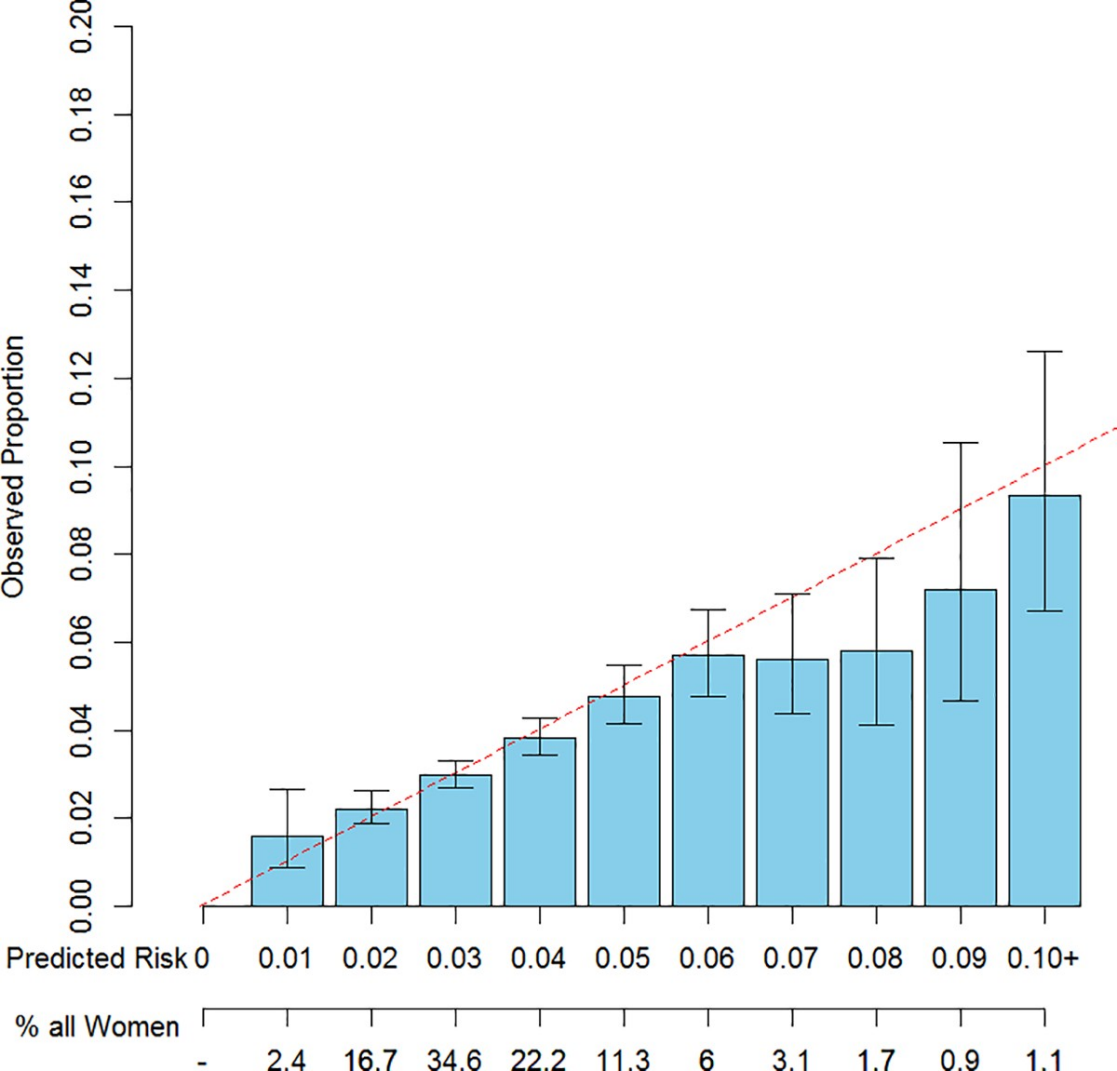
Observed vs. expected counts of 5-year absolute risks vs. 1% risk bins, of 10-fold cross-validated predicted risks using Model 3b.

Figs 2–4 provide visual representations of fitted vs. observed 5-year risks of breast cancer and exact 95% confidence intervals for the fitted risks, stratified by risk category, fit using ten-fold cross validation. The models displayed were the Gail model (Model 0), and Models 3a and 3b (the “best fitting model,” which included additional covariates). The Gail Model did not adequately predict 5-year risk in this cohort, under-estimating risk for women in low risk bins and overestimating risk for women in higher risk bins. However, refitting the model with the Gail covariates and interactions and additional menopause and BMI information resulted in better fit. Among all pairs of statistics, the models that included the BFHS in addition to the 0/1/>1 score demonstrated better fit, as determined by the goodness-of-fit statistic as displayed in Table 3. Results for all models except Model 0 utilized ten-fold cross-validation to avoid overfitting. As expected, models that included more covariates also tended to fit better. Notably, only Model 3b had a goodness-of-fit statistic above 0.05.

**Table 3:** Receiver operating characteristic (ROC) curve analysis and goodness-of-fit statistics for ten-fold cross-validated models.

	AUC	pAUC (specificity 100%-90%)	Sensitivity (at 90% specificity)	Goodness-of-fit statistic
Model 0	0.5928	0.0085	0.1598	3.16 * 10^−14^
Model 1a	0.5782	0.0082	0.1524	1.22 * 10^−9^
Model 1b	0.5802	0.0086	0.1683	1.14 * 10^−7^
Model 2a	0.5949	0.0095	0.1753	1.16 * 10^−11^
Model 2b	0.5957	0.0095	0.1790	4.47 * 10^−9^
Model 3a	0.5963	0.0093	0.1746	0.0213
Model 3b	0.6974	0.0094	0.1775	0.1108

AUCs, pAUCs (specificity 90%-100%), and sensitivities at 90% specificity for the candidate models and the original Gail model are summarized in Table 3. Though differences between these values were small, models that additionally included the BFHS consistently outperformed those with the three-level score in overall AUC. Given the total number of women at risk for breast cancer, these small differences could have meaningful public health impact. Model coefficients, standard errors, and p-values from the best-fitting model (Model 3b) are provided in Table 4. Direction and relative magnitude of coefficient estimates are consistent with current prediction models [4].

**Table 4:** Hazard ratios for best-fitting risk prediction model (Model 3b).

	*HR*	*95% CI (HR)*	*p-value*
*Age at menarche*			
*7–11*	1.016	(0.911, 1.133)	0.778
*12–13*	Ref	Ref	Ref
*≥14*	0.885	(0.796, 0.985)	0.025
*Previous biopsies*			
*0*	Ref	Ref	Ref
*1*	1.489	(1.156, 1.920)	0.002
*>1*	1.243	(0.962, 1.605)	0.096
*Age at first live birth*			
*<20*	Ref	Ref	Ref
*20–24*	0.875	(0.294, 2.605)	0.811
*25–29*	1.517	(0.489, 4.706)	0.470
*≥30*	3.186	(1.105, 9.919)	0.032
*Nulliparous*	0.951	(0.268, 3.371)	0.938
*Age at baseline*			
*<50*	Ref	Ref	Ref
*≥50*	1.126	(0.899, 1.409)	0.301
*Family history (1st-deg. female relatives)*			
*0*	Ref	Ref	Ref
*1*	1.211	(0.527, 2.787)	0.652
*>1*	1.162	(0.472, 2.861)	0.743
***Bayesian Family History Score (BFHS)***	**7.604**	**(1.691, 34.19)**	**0.008**
*BMI category*			
*<25*	Ref	Ref	Ref
*25–30*	1.111	(1.004, 1.229)	0.041
*≥30*	1.203	(1.083, 1.336)	0.001
*Menopause status at baseline*			
*Pre-menopausal*	Ref	Ref	Ref
*Post-menopausal*	0.800	(0.693, 0.924)	0.002
*Interaction: Previous biopsies by Baseline age ≥50*			
*1*	0.795	(0.596, 1.061)	0.120
*>1*	1.274	(0.965, 1.682)	0.088
*Interaction: Age at first live birth by 1 BC+ family history*			
*20–24*	1.170	(0.387, 3.536)	0.781
*25–29*	0.628	(0.199, 1.979)	0.427
*> = 30*	0.369	(0.125, 1.084)	0.070
*Nulliparous*	1.041	(0.289, 3.750)	0.951
*Interaction: Age at first live birth by >1 BC+ family history*			
*20–24*	1.068	(0.349, 3.272)	0.908
*25–29*	0.793	(0.248, 2.533)	0.695
*≥30*	0.473	(0.158, 1.412)	0.179
*Nulliparous*	1.674	(0.460, 6.094)	0.434

**Fig 5:**
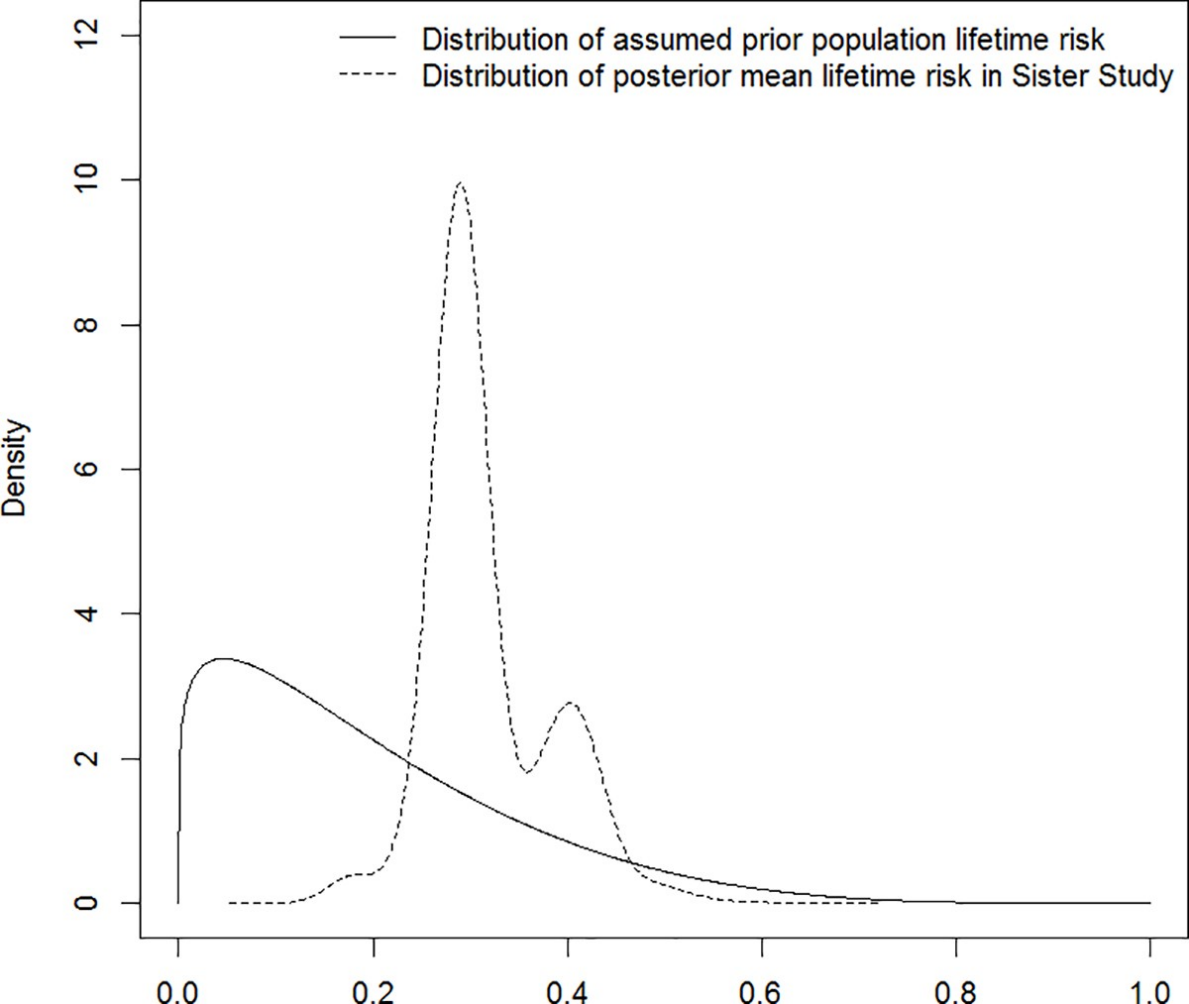
Distribution of assumed prior population pure lifetime breast cancer risk vs. the distribution of posterior mean pure lifetime breast cancer risk based on families participating in the Sister Study cohort.

Finally, the BFHS itself has a natural interpretation as the posterior family-specific lifetime pure breast cancer risk. Fig 5 shows an estimate of this distribution among Sister Study participants, which enables comparison with our assumed beta prior distribution for population risk based on SEER data and epidemiological assumptions. Reflecting the selective sampling used to form the Sister Study cohort, increased information gained through family history alone allows estimation of the upwardly shifted distribution. The mean of the population-based beta distribution was 0.19, while the mean of the kernel estimate for families in the Sister Study is 0.32, suggesting an almost two-fold increase in risk in this cohort of women with first-degree family history of breast cancer, consistent with prior literature.

## Discussion

Our proposed risk score captures detailed information about family structure and history vis-à-vis breast cancer. When applied to a large cohort of women with a first-degree family history of breast cancer, likelihood ratio tests showed that our score provided additional predictive information to a model that used the three-level family history score, both with and without inclusion of known risk factors. Hence, we emphasize the importance of collecting this family history information for population screening purposes and urge the research community to do so in future studies.

An advantage of the Bayesian score is its foundation in likelihood-based methods that take into consideration observed and expected cases among families. Other scores based on observed and expected cases have been proposed: Yang et al. proposed a standardized statistic that aimed to characterize deviations from expected risk for each family; Brewer et al. proposed a standardized incidence ratio as a family history score [17,18]. However, these scores appear somewhat *ad hoc* in their formulation and do not have the benefit of being likelihood-based to lead to a directly interpretable equivalence to family-specific lifetime risk. Rieger and Mansmann have also taken a Bayesian approach in the context of risk modeling in colon cancer in order by treating family history as a Bayesian network modeled using factor graphs [19]. Similarly to our motivation, their family history-based method aims to holistically summarize disparate effects regardless of genetic or environmental mechanism. Unlike our proposed methodology, their approach only models the probability of being a “high risk” family, not the family-specific lifetime risk of disease.

Although one could use the Bayesian score as a multiplier to the estimated SEER population hazard to directly estimate age-specific risk, use of the Bayesian score as a covariate in a Cox model allows incorporation of additional covariate effects. Such covariates may vary through time—due to the nature of the BFHS, it may be constructed as a time-varying covariate itself, in addition to other potentially time-varying covariates such as menopause status. An advantage of the Cox model is the flexible way in which such time-varying covariates can be used to inform risk prediction.

In our analysis, we focused on non-Hispanic white women with a family history of breast cancer, and hence estimated hazard ratios from our model are not immediately generalizable to the wider population. Our analysis results suggested that BFHS is useful in improving accuracy of breast cancer screening in women with family history of breast cancer. However, the proposed score and methodology is generalizable to wide populations: all that is needed is an estimate of the baseline hazard for the population of interest. This can be either estimated using empirical data at hand (e.g., with an existing study), or by using population estimates (e.g., from national registries such as the SEER program). Although estimated hazard ratios may differ, the underlying methodology remains the same. Our approach may also be applied to any disease where family history is an important risk factor and for which necessary surveillance data exist, such as in the previously mentioned application of colon cancer [19].

One limitation is that our proposed score only uses family history of first-degree female relatives, even though information from second-degree relatives may be informative as well. On the other hand, cancer in more distant relatives may be both biologically less informative and less well-reported, detracting from the usefulness of this addition. Though self-reporting error is an issue inherent in any family history-based risk assessment score, we feel that use of information only from first degree female relatives mitigates the recall error problem. Additionally, as long as the misreporting error is only in the ages and not in the status of family members, the impact to the calculated BFHS and therefore risk prediction is relatively minor. To provide a concrete example, suppose a woman aged 51 has three first-degree female relatives, two of them breast cancer free at ages 78 and 53, and one who was diagnosed with cancer at age 55. Suppose the participant misremembers her sister’s diagnosis age and reports 52. This misreporting results in BFHS changing from 0.2844 to 0.2861, which corresponds to the hazard changing to 1.013 times that for the correct family structure when using our best fitting model (3b). Suppose she additionally misremembers one relative’s age as 76 instead of 78. The BFHS changes to 0.2863, which corresponds to hazard approximately 1.015 times that for the correct family structure when using our best fitting model (3b). Since it is unlikely that a woman will misreport the *status* of first-degree female relatives, we believe that the practical impact of misreporting error will be minor (especially as BFHS is only one of many covariates used for risk prediction purposes).

From a practical standpoint, although the AUC in Model 3b of 0.70 may seem to be relatively low *prima facie*, we believe that achieving such an AUC using only family history and a few additional covariates–in the absence of detailed genetic typing–is impressive for a disease with as varied an etiology and risk factors as breast cancer. It is our intention that out score, which only uses information that is relatively easy and accurate to obtain, may be useful to calculate as a simple “first line calculation” that is more accurate than is currently available. Use of a predictive model with our Bayesian score may provide a woman with a general sense of her breast cancer risk and so she can make a more informed decision regarding whether to go for more personalized genetic testing or screening by an expert.

Although women in high risk bins comprise a relatively small portion of the population, the total number of women in the U.S. population who could potentially be assigned to these bins may be large. Thus, improved identification of high risk women is important in minimizing the total health care burden. Still, our proposed score and prediction model apply to women regardless of family history. Unfortunately, our ability to evaluate the performance of our risk prediction approach in very low risk women was limited, because most Sister Study participants are at elevated risk by design. However, our proposed score does have the ability to differentially categorize women at low risk based on family structure. For example, compared to a woman from a larger family with older female relatives without breast cancer, a woman from a small family with younger and as yet cancer-free relatives will have a higher Bayesian score, a subtlety missed in conventional family history scores.

The biggest improvements in risk prediction were in the highest and lowest risk groups. Since the group of women at relatively low risk is a much larger group of women in the general population, use of our proposed score may lead to reduced screening costs, reduced false positive rates, and lower healthcare burden due to unnecessary preventative screening and over-diagnosis. Because we could not directly evaluate performance in low risk women, we explored using existing cohorts focusing on breast cancer as a primary outcome. Unfortunately, most studies have not collected the family history data required (current age or age at death and age at disease onset of first-degree female relatives). Due to potential cost-benefits in the low risk population, validation in other population-based cohorts is recommended.

In summary, detailed first-degree family history data may be considered as a combined surrogate for genetics, lifestyle, and shared environment, and can be easily assessed and updated through questionnaires. Compared to models based on genetics and full pedigree, information needed for calculating our score is easy and inexpensive to obtain. Currently used categorical family history variables lose valuable information that is captured by the Bayesian score. We recommend that future studies collect more detailed first-degree family history data and make use of that information in risk prediction models.

## Supporting information

S1 Fig Illustrative Lexis diagram. (PDF)

S1 Appendix Derivation of bayesian family history score. (DOCX)

S2 Appendix Calculation of 5-year risks. (DOCX)
