# Supplementary figures and images for: Use of detailed family history data to improve risk prediction,with application to breast cancer screening
Source: PLoS One. 2019 Dec 17;14(12):e0226407. doi: 10.1371/journal.pone.0226407 (PMC6917296; doi:10.1371/journal.pone.0226407)

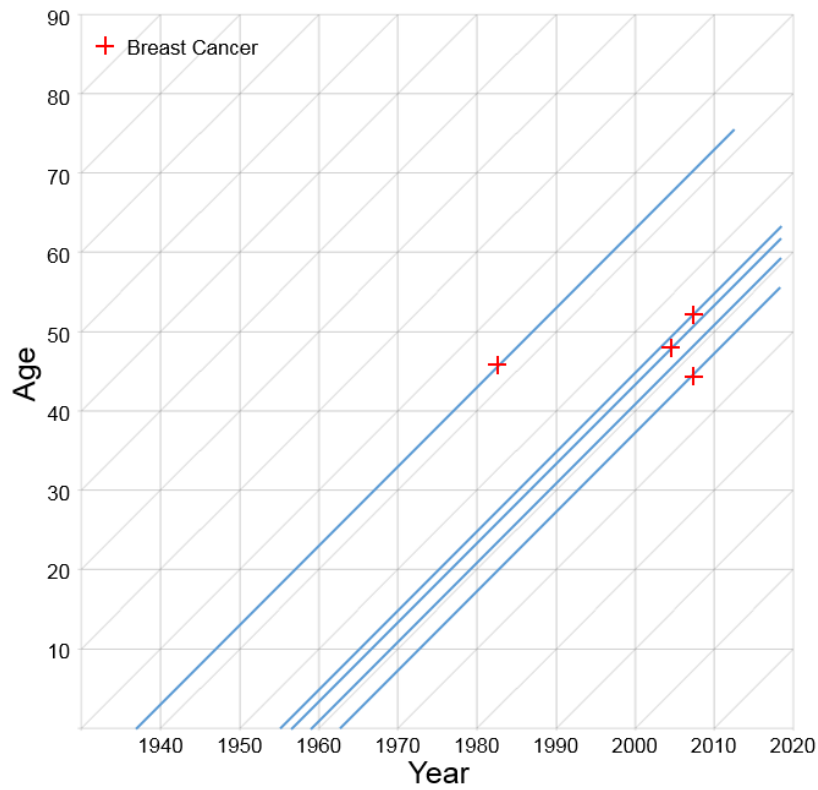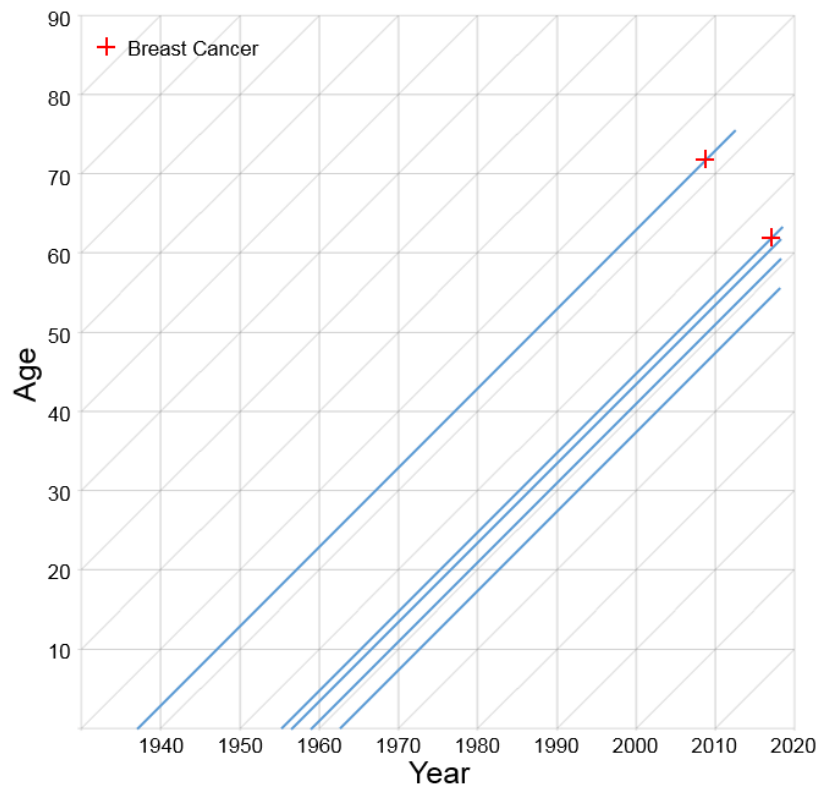

Supplement: S1 Fig — (PDF) [file pone.0226407.s001.pdf]
