# Supplementary material for: Use of detailed family history data to improve risk prediction,with application to breast cancer screening
Source: PLoS One. 2019 Dec 17;14(12):e0226407. doi: 10.1371/journal.pone.0226407 (PMC6917296; doi:10.1371/journal.pone.0226407)
Supplement: S1 Appendix — (DOCX) [file pone.0226407.s002.docx]

**S1 Appendix: Derivation of Bayesian Family History Score**

For each individual in a family, we assume the standard right-censored survival data structure, where the failure event is defined as diagnosis of breast cancer. Let 𝑇 be the age at which she develops breast cancer, $C$ the age at which she is censored, $X =\max(T, C)$ the event age, and $d= 1_{(T<C)}$ the censoring indicator (such that $d=1$ corresponds to diagnosis of breast cancer and $d=0$ corresponds to censoring).

We assume that there is a family-specific lifetime pure breast cancer risk $p$ (conditional on not dying of something else prior to getting breast cancer, and integrating up to age 80) that applies to all women in a specific family. Our goal is to estimate this family-specific lifetime risk, which we will use as a covariate in a Cox proportional hazards model to estimate a woman’s risk of developing breast cancer. We further assume a population hazard function for developing breast cancer $\lambda_{0}(t)$, satisfying the following relation with the population lifetime pure risk (the risk from age 0 to maximum age $U=80$) of breast cancer $p_{0}$:

$$p_{0}=1-exp(-\int_{0}^{U} \lambda_{0}\left( t \right)dt)$$

$$\log\left( 1-p_{0} \right)= -\int_{0}^{U} \lambda_{0}\left( t \right)dt$$

Finally, we assume that each family has a pure lifetime risk p and a function $f\left( p \right)$ that has a multiplicative effect on the hazard function: $\lambda_{p}=f\left( p \right)\lambda_{0}\left( t \right)$. With these assumptions, we have

$$p=1-exp(-\int_{0}^{U} \lambda_{p}\left( t \right)dt)$$

$$=1-exp(-f\left( p \right)\int_{0}^{U} \lambda_{0}\left( t \right)dt)$$

$$\log\left( 1-p \right)= -f\left( p \right)\int_{0}^{U} \lambda_{0}\left( t \right)dt$$

$$f\left( p \right)=\frac{log(1-p)}{log(1-p_{0})}.$$

To estimate the population hazard function to create the Bayesian Family History Score, we use data from the SEER Registries as described in the main manuscrcipt. We approximate the hazard by using one-year breast cancer rates, calculated by performing a fourth-degree polynomial regression of the five-year breast cancer rates across 19 age groups defined by SEER. Plugging in each age into this regression creates a smoothed curve which closely matches raw 1-year rates as provided by SEER*Stat software. The baseline rates used are a sum of the smoothed rates for invasive and *in situ* cases and are pure risks, which do not take into consideration the risk of death.

We assume *a priori* that the baseline lifetime risk for developing breast cancer $p$ follows a $Beta\left( \alpha, \beta\right)$ distribution with expectation $E\left( p \right)$ equal to the pure lifetime risk of invasive and *in situ* cancer calculated from the approximated smoothed hazard function. This expectation is 0.197, which exceeds the combined absolute risk estimate of 0.150 for invasive + *in situ* cases, as expected. From prior knowledge, we make the assumption that the lifetime pure risk of developing breast cancer is doubled given that an individual’s first-degree relative has breast cancer [8,9]. This assumption is supported in our cohort, with the observed/expected case ratio equal to 1.82 where the expected number of cases through our follow-up time was determined using SEER hazards, matching by age and race.

We further assume conditional independence of developing breast cancer among members of a family, and make a simplifying assumption of having only a mother and single full sister. This assumption is based on assuming that on average each child had two siblings, and assuming equal chance of having a sister and a brother. With these assumptions, we have the following calculation:

$$\Pr\left( BCa+ | mother and sister BCa- \right)=\frac{E\left( p\left( 1-p \right)^{2} \right)}{E\left( \left( 1-p \right)^{2} \right)}$$

$$= \frac{\frac{B\left( \alpha+1, \beta+2 \right)}{B\left( \alpha, \beta\right)}}{\frac{B\left( \alpha, \beta+2 \right)}{B\left( \alpha, \beta\right)}}$$

$$=\frac{\Gamma\left( \alpha+1 \right)\Gamma\left( \beta+2 \right)\Gamma\left( \alpha+\beta+2 \right)}{\Gamma\left( \alpha+\beta+3 \right)\Gamma\left( \alpha\right)\Gamma\left( \beta+2 \right)}$$

$$=\frac{\alpha}{\alpha+\beta+2}.$$

$$\Pr\left( BCa+ | at least one BCa+ \right)=\frac{E\left( p\left( 1-\left( 1-p \right)^{2} \right) \right)}{E\left( 1-\left( 1-p \right)^{2} \right)}$$

$$= \frac{E\left( p \right)-E\left( p\left( 1-p \right)^{2} \right)}{1-E\left( \left( 1-p \right)^{2} \right)}$$

$$=\frac{\frac{\alpha}{\alpha+\beta}-\frac{\Gamma\left( \alpha+1 \right)\Gamma\left( \beta+2 \right)\Gamma\left( \alpha+ \beta\right)}{\Gamma\left( \alpha+\beta+3 \right)\Gamma\left( \alpha\right)\Gamma\left( \beta\right)}}{1- \frac{\Gamma\left( \alpha\right)\Gamma\left( \beta+2 \right)\Gamma\left( \alpha+ \beta\right)}{\Gamma\left( \alpha+\beta+2 \right)\Gamma\left( \alpha\right)\Gamma\left( \beta\right)}}$$

$$=\frac{\alpha\left( \alpha+\beta+1 \right)- \frac{\alpha\beta(\beta+1)}{\alpha+\beta+2}}{\left( \alpha+\beta+1 \right)\left( \alpha+ \beta\right)-\beta(\beta+1)}.$$

Hence we have:

$$\frac{Pr(BCa+|at least one BCa+)}{Pr(BCa+|no BCa-)}= \frac{\left( \alpha+\beta+1 \right)\left( \alpha+\beta+2 \right)-\beta(\beta+1)}{\left( \alpha+ \beta\right)\left( \alpha+ \beta+1 \right)-\beta(\beta+1)}=2.$$

Additionally assuming that the expected value of the pure lifetime risk of invasive and *in situ* cancer is 0.197, we have the equation

$$\frac{\alpha}{\alpha+ \beta}=0.197.$$

Solving these two equations simultaneously yields $\left( \alpha, \beta\right)$ equal to (1.1845, 4.8281).

Our likelihood is a function of the observed data, which comes from the participant herself and her first-degree female relatives. The likelihood for a specific family depends on the event ages, X_i_, at which female first-degree relatives either died, were diagnosed with breast cancer, or were ascertained to be alive and breast-cancer-free, is given by

$$L\left( p;X,d \right)= \Pi_{i=1}^{n}\left\{ \lambda_{p}\left( X_{i} \right) \right\}^{d_{i}}\{S_{p}\left( X_{i} \right)\}$$

$$= \Pi_{i=1}^{n}\{f\left( p \right)\lambda_{0}{\left( X_{i} \right)\}}^{d_{i}}\exp\left\{ -f\left( p \right)\Lambda_{0}\left( X_{i} \right) \right\}.$$

where S_p_(X_i_) is the probability of not getting breast cancer up to age X_i_ and $\Lambda_{0}\left( X_{i} \right)$ is the integrated hazard up to age X_i_. Thus, our posterior distribution for this specific family of size n: a participating woman and her n-1 first-degree female relatives is given by

$$Pr\left( p;X, d \right)\propto Pr\left( p; \alpha, \beta\right)\times L\left( p;X, d \right)$$

$$=p^{\alpha-1}\left( 1-p \right)^{\beta-1}\Pi_{i}^{n}\{f\left( p \right)\lambda_{0}{\left( X_{i} \right)\}}^{d_{i}}\exp\left\{ -f\left( p \right)\Lambda_{0}\left( X_{i} \right) \right\}$$

$$=p^{\alpha-1}\left( 1-p \right)^{\beta-1}\Pi_{i}^{n}\{f\left( p \right)\lambda_{0}{\left( X_{i} \right)\}}^{d_{i}}\left\{ \left( 1-p \right)^{-\frac{\Lambda_{0}\left( X_{i} \right)}{\log\left( 1-p_{0} \right)}} \right\}$$

$$=p^{\alpha-1}\left( 1-p \right)^{\beta-c-1}\{{\log\left( 1-p \right)\}}^{\sum_{i=1}^{n} d_{i}}\left\{ \frac{\lambda_{0}\left( X_{i} \right)}{\log\left( 1-p_{0} \right)} \right\}^{\sum_{i=1}^{n} d_{i}}$$

$$\propto p^{\alpha-1}\left( 1-p \right)^{\beta-c-1}({\log\left( 1-p \right))}^{\sum_{i=1}^{n} d_{i}},$$

$$\mathrm{where} c= \sum_{i}^{n} \frac{\Lambda_{0}\left( X_{i} \right)}{\log\left( 1-p_{0} \right)}.$$

We take the posterior mean of the family-specific lifetime breast cancer risk $p$, based on family history alone, as our Bayesian Family History Score for this particular family:

$$\hat{p}=E\left( p;X, d \right)= \frac{\int_{0}^{1} p^{\alpha}\left( 1-p \right)^{\beta-c-1}\left( \log\left( 1-p \right) \right)^{\sum_{i=1}^{n} d_{i}}}{\int_{0}^{1} p^{\alpha-1}\left( 1-p \right)^{\beta-c-1}\left( \log\left( 1-p \right) \right)^{\sum_{i=1}^{n} d_{i}}}.$$

We use numerical integration methods (adaptive quadrature) to calculate this expectation. We see that the Bayesian Family History Score depends on $(\alpha, \beta)$ from our prior distribution, $c$, a unique family-specific construct which accounts for both family size and the total risk experienced up to the present time in this family, and the sum of $d_{i}$, the cumulative number of affected first-degree female relatives.
