# Supplementary material for: Use of detailed family history data to improve risk prediction,with application to breast cancer screening
Source: PLoS One. 2019 Dec 17;14(12):e0226407. doi: 10.1371/journal.pone.0226407 (PMC6917296; doi:10.1371/journal.pone.0226407)
Supplement: S2 Appendix — (DOCX) [file pone.0226407.s003.docx]

**S2 Appendix: Calculation of 5-year risks**

For each individual, we can calculate her Bayesian family history score, $\hat{p}$, based on her first-degree relatives’ experience and applying the derivations in Appendix 1. We can also make use of relevant covariates for risk prediction purposes (for instance, Gail model-identified covariates). With model coefficients from a fitted Cox model, we can calculate the linear predictor $X\hat{\beta}$, which is a linear combination of model coefficients and relevant observed data. This linear predictor can be used to calculate 5-year cause-specific (pure) risks as follows for individual $i$:

$$P\left( T\in\left( t, t+5 \right) | X, T>t \right)=1-\exp\left( -\int_{t}^{t+5} \hat{\lambda}_{0}\left( s \right)\exp\left( X\hat{\beta} \right)ds \right),$$

where t is the baseline age from which to make a prediction for individual$i$, $X\hat{\beta}$ is the linear predictor as described above, and $\hat{\lambda}_{0}\left( t \right)$ is the estimated baseline hazard function.

In our Cox models, we ignored all other competing risks, and fitted a cause-specific hazard model to estimate pure risk of breast cancer. In practice, we aim to make apples-to-apples comparisons with the Gail model, which examines absolute risks instead. To approximate absolute risks given only pure risks, we discretize the entire five year interval into one-year sub-intervals, and perform the following procedure:

1. The absolute risk for the first sub-interval is taken to be the pure risk for that interval.
2. The absolute risk for the next sub-interval is the pure risk for that interval, multiplied by the probability that the individual both did not die in the previous interval and did not develop breast cancer in the previous interval.
3. Iterate the second step until the end of the risk prediction period of interest (for instance, four sub-intervals after the first to calculate five-year risk).
4. Sum all calculated absolute risks.

This procedure allows for accurate calculation of absolute risks when only pure risks of the event of interest and of mortality are known, as was confirmed in an illustrative example by Gail [12].

In particular, the absolute and pure risks for each five year interval are given as follows. Let $P_{t}$ be the pure one-year risk of developing breast cancer in the interval $[t, t+1)$. Let $A_{t}$ be the absolute one-year risk of developing breast cancer in that interval. Let $M_{t}$ be the pure one-year risk of death in that interval. We have values for $P_{t}$ and $M_{t}$ for each interval. The absolute risks are calculated as follows:

1. $A_{t}=P_{t}$
2. $A_{t+1}=P_{t+1}\times\left( 1-A_{t}-M_{t} \right)$
3. $A_{t+2}=P_{t+2}\times\left( 1-A_{t+1}-M_{t+1}-A_{t}-M_{t} \right)$
4. $A_{t+3}=P_{t+3}\times\left( 1-A_{t+2}-M_{t+2}-A_{t+1}-M_{t+1}-A_{t}-M_{t} \right)$
5. $A_{t+4}=P_{t+4}\times\left( 1-A_{t+3}-M_{t+3}-A_{t+2}-M_{t+2}-A_{t+1}-M_{t+1}-A_{t}-M_{t} \right)$

Then total absolute risk in the interval is simply the sum of the absolute risks in the five intervals.

The probability of dying in each sub-interval may be based on estimated data from an existing cohort, or from national statistics such as the National Vital Statistics System. We require age-specific rates of dying within 1 year, conditioned on being alive at the start of that age bracket. For sub-intervals that straddle two ages, we sum the fractional component from each age. As an example, to evaluate probabilities in the age sub-interval 58.4 – 59.4, we sum 0.6 times the relevant probabilities for age 58 and 0.4 times the relevant probabilities for age 59. We consider the pure risk of all-cause mortality, including from breast cancer, in the approximation. Though ideally we would use non-breast cancer mortality, the difference is expected to be negligible in the calculation.
